# Novel insights regarding the sigmoidal pattern of resistance to neomycin conferred by the *aphII* gene, in *Streptomyces lividans*

**DOI:** 10.1186/2191-0855-3-13

**Published:** 2013-02-08

**Authors:** Nicolas Seghezzi, Marie-Joelle Virolle, Patrick Amar

**Affiliations:** 1 Institut de Génétique et Microbiologie, UMR8621 CNRS Université Paris-Sud, Orsay, 91405, France; 2 Laboratoire de Recherche en Informatique, Université Paris-Sud, UMR8623 CNRS, and INRIA Saclay, Orsay, 91405, France

**Keywords:** Bacteria, Gene expression/regulation, Growth and survival, Modelling

## Abstract

A library of synthetic promoters of various strengths, specifically constructed for *Streptomyces* species, was cloned in the promoter-probe plasmid pIJ487, upstream of the promoter-less *aphII* gene that confers resistance to neomycin. The survival rates conferred by promoters were assessed in the presence of 100 μg.ml^−1^ neomycin. The correlation between the transcriptional activity of the *aphII* gene (estimated by RT-PCR) and the resistance to neomycin (expressed as survival rate) indicated a sigmoid rather than a linear correlation. In this issue, we propose a tentative explanation for this sigmoidal pattern of resistance in relation with the level of *aph*II gene expression. Beyond this specific example, our model might constitute a sound explanation for the generally observed but never explained sigmoidal shape of classical inhibition curves obtained in the presence of linearly increasing antibiotic concentrations.

## Introduction

Antibiotics have been the most useful therapeutic agents of the twentieth century (Levy 2002). However, more and more bacteria have developed resistance to all existing antibiotics and antimicrobial resistance was recently recognized as one of the greatest threats to human health (Gyssens 2011). Indeed an increasing number of patients suffer from serious life-threatening antimicrobial-resistant infections against which only very few, if any, effective antibiotics are available (Levy 1998). Alarmingly, as the number of patients dying from antibiotic-resistant infections rises, the number of new antibiotics in development is plummeting (Butler and Cooper 2012; Mahajan and Balachandran 2012). The antibiotic resistance genes acquired by human pathogens are thought to originate from micro-organisms of the environment including *Streptomyces* (Forsberg et al. 2012). These bacteria are antibiotic producers and thus contain the corresponding antibiotic resistance genes for self protection (Allen et al. 2010; Davies and Davies 2010; Nikaido 2009). The genes conferring resistance to antibiotics are spreading by horizontal transfer in the microbial population and the release of antimicrobials in the environment likely selects micro-organisms carrying these genes (Alonso et al. 2001; Martinez 2008). The mechanisms conferring antibiotic resistance in micro-organisms include enzymatic inactivation or modification of the antibiotic, modification of host targets to prevent antibiotic binding, efflux pumps. A major challenge to counteract the development of resistance to antibiotic treatment is to get a better understanding of how bacteria react to antibiotics.

The efficiency of many antibiotics is known to be impaired by the existence of resistance mechanisms. In this study, an antibiotic resistance gene, *aph*II, was used to assess the impact of the level of expression of this gene on survival to an antibiotic selective pressure. *aph*II encodes a phosphotransferase, phosphorylating the aminoglycoside antibiotic, neomycin, impairing its ability to interact with the ribosome and thus preventing inhibition of mRMA translation by neomycin (Beck et al. 1982; Hermann 2007). The expression of this gene present on the multicopy plasmid pIJ487 (Ward et al. 1986) was put under the control of 18 promoters of varying strengths originating from a previous study (Seghezzi et al. 2011). These different constructs were introduced into *Streptomyces lividans* and survival of these different clones, exposed to a constant and rather high concentration of neomycin (100 μg/ml), was assessed.

Our results revealed that the relation between promoter strength (as determined by RT-PCR) and survival rate was not linear but indicated a sigmoidal correlation. A model, consistent with this behaviour, based on the well-known mechanism by which aminoglycoside antibiotics are lethal to bacteria, and how AphII counteracts this poisoning activity, was designed and discussed. This model might have a more general scope to rationalize the currently observed but never explained sigmoidal shape of classical inhibition curve obtained with linearly increasing antibiotic concentrations (Baudoux et al. 2007).

## Materials and methods

### Bacterial strains, plasmid and media

*Streptomyces lividans* TK24 strains transformed with 38 pIJ487-derived plasmids, each carrying a 300 bp DNA fragment with promoter activity of various strength were used in this study (Ward et al. 1986). The strength of these promoters was previously roughly estimated, using the replica-plating technique as described in ( Lederberg and Lederberg 1952). These promoters were classified as weak, medium or strong based on their ability to allow growth of the different transformants in the presence of up to 20, 50 or 100 μg.ml^−1^ of neomycin in HT medium (Seghezzi et al. 2011). Media as well as *Streptomyces* manipulations were carried out according to Practical Streptomyces Genetics manual (Kieser et al. 2000). SFM was used to grow up transformants to prepare spores suspensions for quantitative estimation of survival rates.

### Estimation of survival rates

**Figure 1:**
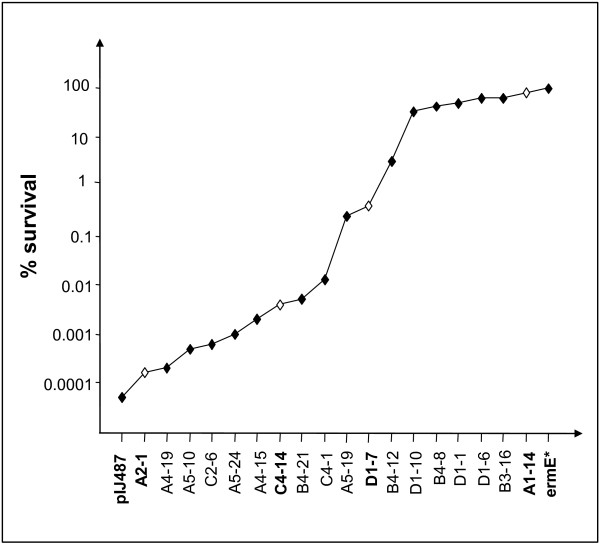
**Survival rates conferred by promoters of different strength on HT medium containing 100 μg.ml**^**-1**^**neomycin.** Promoters strength was initially roughly assessed by patch replica plating on HT medium containing different concentrations of neomycin. Transcriptional activity conferred by the promoters indicated in bold as well as that originating from control plasmids (promoter-less pIJ487 and pIJ487ermE*) were determined by RT-PCR.

Glycerol stocks of spores of the different transformants made on SFM medium were precisely titrated by plating different spore dilutions on HT agar containing 50 μg.ml^−1^ thiostrepton. Subsequently, 10^2^ and 10^3^ spores of each transformant were spread out on HT agar plates containing 50 μg.ml^−1^ thiostrepton only or 50 μg.ml^−1^ thiostrepton and 100 μg.ml^−1^ neomycin. Neomycin resistant colonies were counted after incubation for 72 hours at 30°C and viability rates were calculated relative to the number of colonies on neomycin free plates. These platings, done in duplicate, gave very similar viability counts and the means are shown in Figure 1.

### Estimation of aphII transcriptional activity using RT-PCR

RNA was extracted, using RNeasy Mini Kit from Qiagen, from selected transformants representative of each strength class and grown for 48h on the surface of cellophane disks laid on solid HT medium containing only 50 μg.ml^−1^ thiostrepton.

RT PCR was performed using the OneStep RT-PCR Kit from Qiagen and the following conditions: initial denaturation at 97°C, 5 min followed by 25 cycles of denaturation (97°C, 30 s), annealing (50°C, 30 s) and extension (72°C, 30 s). The absence of DNA in RNA samples was systematically checked by running a control PCR reaction made in absence of reverse transcription. Quantification of the RT-PCR signals was made using ImageQuant pixel counts in non-saturated conditions. Values were normalised on pIJ487 signals. The normalisation was done with the negative control that is the plasmid pIJ487 with no promoter cloned upstream of *aph*II. Some weak transcription of *aph*II was detected in this context.

## Results

### Assessment of promoter strength using viability assays and RT-PCR

An accurate quantification of promoter strength, expressed as survival rate, was carried out in the presence of 100 μg.ml^-1^ neomycin, for 38 selected clones belonging to a previously constructed bank of synthetic promoter designed for *Streptomyces* species and fused to the reporter gene *aphII* conferring resistance to neomycin. These promoters were previously roughly classified as weak, medium and strong by replicate plating (Seghezzi et al. 2011). A transformant containing pIJ487ermE* carrying the strong ermE* promoter was used as a positive control (Bibb et al. 1985). It should be stressed that in a genetically homogenous bacterial population, all the bacteria are not in the same physiological state and the expression of *aphII* (as that of any other genes) varies stochastically around a mean value (Elowitz et al. 2002; Losick and Desplan 2008). This variability explains why, even when a weak promoter is driving *aphII* expression, a small fraction of the bacterial population is able to resist to a high level of neomycin.

Eighteen of these transformants, representative of each class of promoter strength (weak, medium and strong) were precisely ranked according to the survival rate they conferred in the presence of 100 μg.ml^-1^ neomycin. All colonies had approximately the same size. Each promoter was plotted against the log of the survival rate it conferred in the presence of 100 μg.ml^-1^ neomycin. Interestingly, the resulting curve appears to be sigmoidal (Figure 1). The first and third parts represent the low and high survival rates and the central part of the curve shows an abrupt transition between these two states. We thus wondered whether the transcriptional activity of these promoters followed a similar sigmoidal pattern. To answer this question, we assessed the transcriptional activity of promoters corresponding to the three parts of the curve, using RT-PCR.

**Figure 2:**
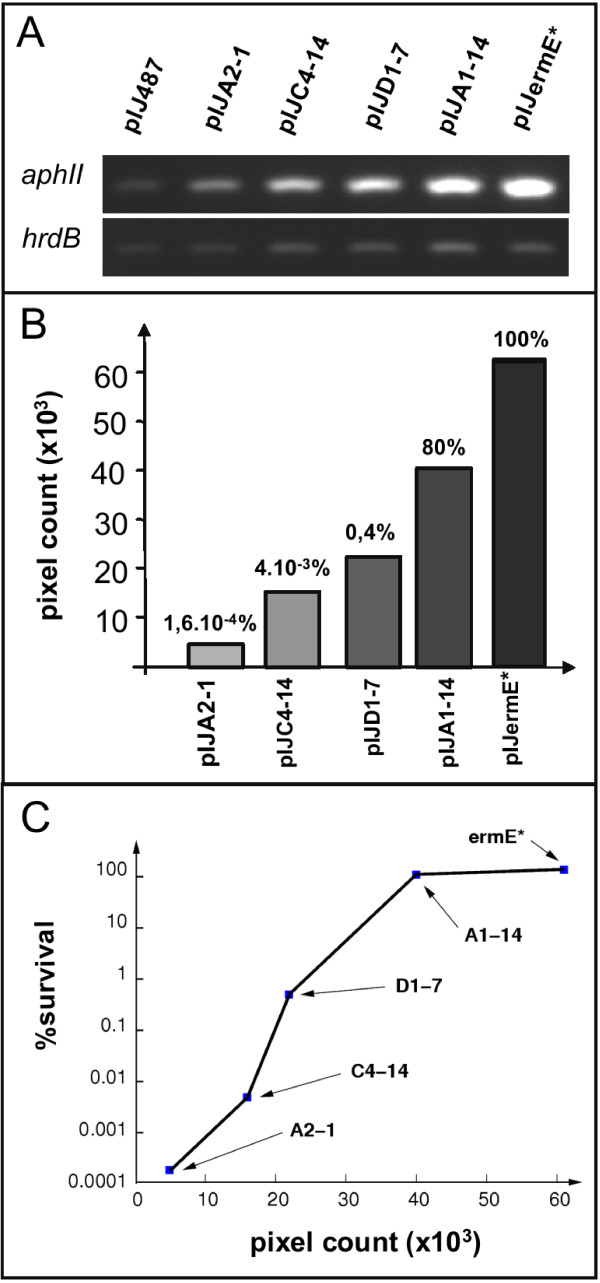
**Correlation between the level of transcription of the *aphII *gene and the survival rates of different transformants in the presence of 100 μg.ml**^**-1**^**neomycin.** (**A**) Assessment of the transcriptional activity of *aphII* in *S. Lividans* driven by the promoters highlighted in bold in Figure 1 and the control plasmids, pIJ487 and pIJ487ermE* , using RT-PCR. Clones were ordered from the left to the right according to increasing promoter strength. (**B**) Quantification of the RT-PCR signals was made using ImageQuant pixel counts in non-saturated conditions. Values were normalized on pIJ487 signals. Survival rates corresponding to each clone are indicated above each histogram. (**C**) Plotting of the quantification of the RT-PCR products of the different clones versus their corresponding survival rates in presence of 100 μg.ml^-1^ neomycin.

Results shown in the Figure 2AB indicated that the transcriptional activity between the weakest and the strongest promoter was approximately 12 fold whereas the corresponding overall increase in the survival rate was in the 10^6^ fold range. Similarly, the two fold increase in the level of expression between the D1-7 and A1-14 promoters led to a 200 fold increase in the survival rate. The level of gene expression was thus plotted versus the survival rates and this plotting (Figure 2C) indicated a non-linear, roughly sigmoidal, correlation between the level of gene expression and the survival rate. We thus proposed an explanatory model that rationalises the sigmoidal pattern of resistance to neomycin observed in relation with the level of *aph*II gene expression and with the concentration of neomycin used.

#### The model

**Figure 3:**
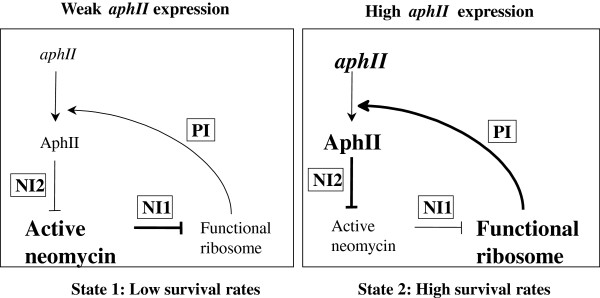
**Schematic representation of the two states of the system.** State 1: Low survival rates: If the expression of *aphII* is low, AphII is not abundant and most ribosomes are poisoned. This poisoning leads to a further reduction of AphII abundance and thus to low survival rates. State 2: High survival rates: If the expression of *aphII* is high, AphII is abundant, more neomycin is inactivated so less ribosomes are poisoned and high survival rates are observed. A small variation of *aphII* expression around a specific threshold leads to an abrupt switch from one state to the other.

Our study revealed a sigmoidal correlation between the level of *aph*II gene expression and the survival rates, in the presence of neomycin 100 μg.ml^-1^ (Figure 2C). This behaviour is reminiscent of a system containing a positive feedback loop leading to two different states, the transition between the two states being very abrupt (Kaufman et al. 2007; Mehra et al. 2008; Mitrophanov and Groisman 2008). In our case, the positive feedback loop would be constituted by one positive and two negative interactions (Figure 3). Positive interaction (PI): the ribosomes are involved in protein synthesis. Negative interaction (NI1): neomycin poisons the ribosomes leading to an inhibition of protein synthesis, including that of AphII. Negative interaction (NI2): AphII inactivates neomycin. These two negative interactions and the positive one generate a positive circuit. When the expression of *aphII* is low, AphII is not very abundant so most of neomycin is active and a lot of ribosomes are poisoned. This poisoning leads to a further reduction of AphII and thus to low survival rates (State 1). Conversely, when the expression of *aphII* is high, AphII is abundant, most neomycin is inactivated so more ribosomes are functional leading to a further enhancement of *aph*II mRNA translation leading to high survival rates (State 2). A small variation of gene expression around a specific threshold leads to an abrupt switch from one state to the other. Experimentally, the level of expression of the D1-7 promoter was shown to be close to this threshold.

## Discussion

The *aphII* gene is the most extensively used reporter system in *Streptomyces*, however, this useful system was sometimes blamed for some non-understood paradoxical behaviour. The dynamics of the system, as revealed by our study, can explain these paradoxes. Consequently, the *aphII* gene should thus be used with caution to accurately assess promoter strength, since a small variation of gene expression around a specific threshold might lead to a huge change in the pattern of resistance to neomycin.

Furthermore, our study suggests that it might be sufficient to reduce (and not totally preclude) the expression of a resistance gene just under a certain threshold to greatly enhance the killing efficiency of the corresponding antibiotic. That is why nowadays even imperfect inhibitors of transcription and/or translation are sometimes associated in prescription to overcome some reluctant antibiotic resistant strains.

**Figure 4:**
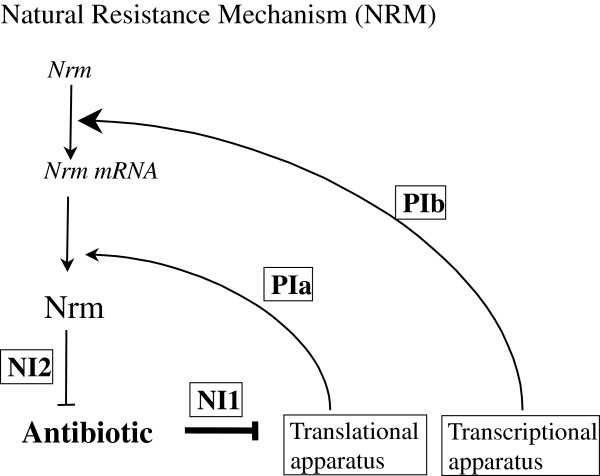
**Model of natural mechanisms of resistance against antibiotics targeting the transcriptional and/or the translational apparatus.** The transcriptional and the translational apparatus are involved in protein synthesis (positive interactions PIa, PIb). The lethal effect of an antibiotic relies on its ability to inhibit transcription or translation (first negative interaction, NI1) including that of a gene encoding a protein neutralizing somehow the poisoning effect of the antibiotic (second negative interaction, NI2). The positive and the two negative interactions generate a positive circuit that leads to the establishment of a sigmoidal pattern of resistance to the antibiotic according to the level of expression of the gene responsible for the natural resistance mechanism.

At last, it is noteworthy that the sigmoidal shape of our curve is reminiscent of that of classical inhibition curve obtained with linearly increasing concentration of antibiotics (Baudoux et al. 2007; Mattie 2000). This similarity suggests that related processes might take place in a natural strain. Any natural strain, in which no exogenous antibiotic resistance gene was introduced, does possess more or less efficient active antibiotic resistance processes. These processes might include efflux of the antibiotic *via* multidrug efflux pumps ( Nikaido 2009), built-in target (Criswell et al. 2006; Long et al. 2009) or antibiotic modifications or proteic systems that might counteract the detrimental consequences induced by the antibiotic such as the generation of oxidative stress etc. ( Hassett and Imlay 2007; Kohanski et al. 2007; Shin et al., 2011). Since all these resistance processes involve genes that should be transcribed and mRNA that should be translated, it is not unreasonable to think that any antibiotic targeting the transcriptional or translational apparatus would give rise to a positive circuit resulting in a sigmoidal response (Figure 4). Our model thus might provide a sound explanation for the sigmoidal shape of the inhibition curves that has been repeatedly observed but was never explained.

## Competing interests

The authors declare that they have no competing interests.
